# Altered basal lipid metabolism underlies the functional impairment of naive CD8^+^ T cells in elderly humans

**DOI:** 10.4049/jimmunol.2100194

**Published:** 2022-01-14

**Authors:** Francesco Nicoli, Mariela P. Cabral-Piccin, Laura Papagno, Eleonora Gallerani, Mathieu Fusaro, Victor Folcher, Marion Dubois, Emmanuel Clave, Hélène Vallet, Justin J. Frere, Emma Gostick, Sian Llewellyn-Lacey, David A. Price, Antoine Toubert, Loïc Dupré, Jacques Boddaert, Antonella Caputo, Riccardo Gavioli, Victor Appay

**Affiliations:** a Centre d’Immunologie et des Maladies Infectieuses (CIMI-Paris), Sorbonne Université, INSERM U1135, 75013 Paris, France; b Department of Chemical, Pharmaceutical and Agricultural Sciences, University of Ferrara, Ferrara 44121, Italy; c Toulouse Institute for Infectious and Inflammatory Diseases (INFINITy), Université Toulouse III, INSERM UMR1291 / CNRS UMR5051, 31300 Toulouse, France; d Institut de Recherche Saint Louis, EMiLy, Université de Paris, INSERM U1160, 75010 Paris, France; e Service de Gériatrie, Hôpital Pitié-Salpêtrière, AP-HP, 75013 Paris, France; f Department of Immunobiology and the Arizona Center on Aging, University of Arizona College of Medicine Tucson, Tucson, AZ 85724, USA; g Division of Infection and Immunity, Cardiff University School of Medicine, Cardiff CF14 4XN, UK; h Systems Immunity Research Institute, Cardiff University School of Medicine, Cardiff CF14 4XN, UK; i Laboratoire d’Immunologie et d’Histocompatibilité, Hôpital Saint-Louis, AP-HP, 75010 Paris, France; j Ludwig Boltzmann Institute for Rare and Undiagnosed Diseases (LBI-RUD), 1090 Vienna, Austria; k Department of Dermatology, Medical University of Vienna, 1090 Vienna, Austria; l International Research Center of Medical Sciences, Kumamoto University, Kumamoto 860-0811, Japan

**Keywords:** Aging, fatty acids, immunosenescence, naive T cells

## Abstract

Aging is associated with functional deficits in the naive T cell compartment, which compromise the generation of *de novo* immune responses against previously unencountered antigens. The mechanisms that underlie this phenomenon have nonetheless remained unclear. We found that naive CD8^+^ T cells in elderly humans were prone to apoptosis and proliferated suboptimally in response to stimulation via the TCR. These abnormalities were associated with dysregulated lipid metabolism under homeostatic conditions and enhanced levels of basal activation. Importantly, reversal of the bioenergetic anomalies with lipid-altering drugs, such as rosiglitazone, almost completely restored the antigen responsiveness of naive CD8^+^ T cells. Interventions that favor lipid catabolism may therefore find utility as adjunctive therapies in the elderly to promote vaccine-induced immunity against targetable cancers and emerging pathogens, such as seasonal influenza viruses and SARS-CoV-2.

## Introduction

Life expectancy has increased considerably over the last century as a consequence of advances in medicine and improved public health systems. However, old age is associated with a high prevalence of chronic diseases and an increased susceptibility to cancer and emerging pathogens, such as SARS-CoV-2 (1). Age-related deficits in the immune system are thought to play a key role in the development of many pathological conditions (2–4). Immune aging is characterized by a progressive erosion of the naive CD8^+^ T cell compartment, which impairs *de novo* immune responses against newly encountered antigens (5–7). Alongside this decline in absolute numbers (8), naive CD8^+^ T cells in elderly individuals also exhibit various functional limitations (9), including suboptimal differentiation in response to T cell receptor (TCR)-mediated activation (5).

A growing body of evidence indicates that lymphocyte metabolism is a key determinant of immune functionality (10–13). Systemic metabolic disturbances are common in elderly individuals, and increased levels of adipokines and proinflammatory lipid species in particular have been implicated as critical mediators of inflammaging, which is thought to exacerbate many age-related diseases (14). In this study, we investigated the bioenergetic features of naive CD8^+^ T cells in middle-aged and elderly humans, aiming to establish a link between metabolic disturbances and immunosenescence. Naive CD8^+^ T cells displayed functional and metabolic abnormalities in elderly people, including enhanced lipid influx and storage accompanied by a marked susceptibility to apoptosis and impaired proliferation in response to activation via the TCR. Importantly, these deficits were mitigated in the presence of lipid-altering drugs, opening potential therapeutic avenues to enhance immune reactivity in later life.

## Material and Methods

### Donors and samples

Two groups of healthy volunteers were enrolled in this study: (i) middle-aged Caucasians (median, 39 years; range, 19–55 years); and (ii) elderly Caucasians (median, 82 years; range 65–95 years). Individuals with malignancies, acute diseases, or severe chronic diseases, such as atherosclerosis, congestive heart failure, poorly controlled diabetes mellitus, renal or hepatic disease, various inflammatory conditions, or chronic obstructive pulmonary disease, as well as individuals on immunosuppressive therapy, were excluded from the study. Laboratory staff were blinded to donor identity. Ethical approval was granted by the Comité de Protection des Personnes of the Pitié Salpétrière Hospital (Paris, France). All volunteers provided written informed consent in accordance with the principles of the Declaration of Helsinki. PBMCs were isolated from venous blood samples via density gradient centrifugation according to standard protocols and cryopreserved in complete medium supplemented with DMSO (10% v/v; Sigma-Aldrich) and FCS (20% v/v; Sigma-Aldrich). Complete medium (R+) consisted of RPMI 1640 supplemented with non-essential amino acids (1% v/v), penicillin/streptomycin (100 U/mL), L-glutamine (2 mM), and sodium pyruvate (1 mM) (all from Thermo Fisher Scientific).

### Flow cytometry and cell sorting

PBMCs were stained for surface markers using combinations of the following directly conjugated monoclonal antibodies: anti-CCR7–BV650 (clone 3D12; BD Biosciences), anti-CCR7–PE-Cy7 (clone 3D12; BD Biosciences), anti-CD3–BV605 (clone SK7; BD Biosciences), anti-CD8–APC (clone RPA-T8; BD Biosciences), anti-CD8–APC-Cy7 (clone SK1; BD Biosciences), anti-CD8–FITC (clone RPA-T8; BD Biosciences), anti-CD27–AF700 (clone O323; BioLegend), anti-CD27–PE (clone M-T271; BD Biosciences), anti-CD45RA–ECD (clone 2H4LDH11LDB9; Beckman Coulter), anti-CD45RA–PerCP-Cy5.5 (clone HI100; eBioscience), anti-CD45RA–V450 (clone HI100; BD Biosciences), anti-CD49b–PE-Cy7 (clone 9F10; BioLegend), anti-CD57– Pacific Blue (clone HCD57; BioLegend), and anti-CD95–FITC (clone DX2; BD Biosciences). Naive CD8^+^ T cells were defined as CD3^+^ CD8^+^ CD27^+^ CD45RA^+^ CCR7^+^ in most experiments and further identified as CD49b^–^ CD57^–^ CD95^–^ for gene expression studies and intracellular measurements of T-bet. Non-viable cells were eliminated from the analysis using LIVE/DEAD Fixable Aqua (Thermo Fisher Scientific). Intracellular stains were performed using anti-T-bet–eFluor660 (clone 4B10; eBioscience) in conjunction with a Transcription Factor Buffer Set (BD Biosciences). Samples were acquired using an LSR Fortessa or a FACSCanto II (BD Biosciences). Naive CD8^+^ T cells were flow-sorted using a FACSAria II (BD Biosciences). Data were analyzed using FACSDiva software version 7 (BD Biosciences) and/or FlowJo software version 10 (FlowJo LLC).

### Proliferation assays and cell culture conditions

PBMCs were labeled with Cell Proliferation Dye (CPD) eFluor450 (Thermo Fisher Scientific) and stimulated for 4 days with plate-bound anti-CD3 (clone OKT3; Thermo Fisher Scientific). In some experiments, cells were precultured in AIM-V medium (Thermo Fisher Scientific) supplemented with BSA (10% v/v; Sigma-Aldrich) for 1 day in the absence or presence of palmitic acid (PA; 300 μM; Sigma-Aldrich), and in other experiments, cells were precultured in AIM-V medium (Thermo Fisher Scientific) without BSA supplementation for 2 days in the absence or presence of rosiglitazone (40 μM; Sigma-Aldrich). Proliferation was measured using flow cytometry to quantify the dilution of CPD. Sample size calculation, based on available data for T-cell proliferation (5), suggested a size of 10 individuals per group to detect a 50% difference in CPD low (i.e. proliferating) cells between middle-aged and old individuals with a power of 80% using a one-sided significance level of 5%. To assess the effect of fatty acids on resting cells, PBMCs were cultured up to 48 hr in AIM-V medium supplemented with 10% BSA in the absence or in the presence of palmitic acid (300 or 3000 μM).

### Activation assays

PBMCs were stimulated for 24 hr with plate-bound anti-CD3 (clone OKT3; Thermo Fisher Scientific) in the absence or presence of fenofibrate (50 μM; Sigma-Aldrich) or rosiglitazone (40 μM; Sigma-Aldrich). Activation markers were quantified on the cell surface using anti-CD69–FITC (clone L78; BD Biosciences) and anti-CD134–BV711 (clone ACT35; BD Biosciences). Intracellular stains were performed using anti-active caspase-3–PE (clone C92-605; BD Biosciences) in conjunction with a Cytofix/Cytoperm Fixation/Permeabilization Solution Kit (BD Biosciences).

### Metabolism assays

To determine glucose uptake, PBMCs were incubated for 20 min at 37°C in PBS containing 2′-(N-(7-nitrobenz-2-oxa-1,3-diazol-4-yl)amino)-2-deoxyglucose (2-NBDG; 50 μM; Thermo Fisher Scientific). To determine fatty acid (FA) uptake, PBMCs were incubated for 20 min at 37°C in PBS containing 4,4-difluoro-5,7-dimethyl-4-bora-3a,4a-diaza-*s*-indacene-3-hexadecanoic acid (BODIPY FL C16; 1 μM; Thermo Fisher Scientific). To determine neutral lipid (NL) content, PBMCs were incubated for 20 min at 37°C in PBS containing 4,4-difluoro-1,3,5,7,8-pentamethyl-4-bora-3a,4a-diaza-*s*-indacene (BODIPY 493/503; 10 μM; Thermo Fisher Scientific). To determine mitochondrial mass, PBMCs were incubated for 30 min at 37°C in R+ containing Mitotracker Deep Red (500 nM; Thermo Fisher Scientific). To determine mitochondrial membrane potential (ΔΨM), PBMCs were incubated for 30 min at 37°C in R+ containing tetramethylrhodamine, methyl ester, perchlorate (TMRM; 25 nM; Thermo Fisher Scientific). To determine mTOR activity, PBMCs were incubated for 10 min at 37°C in Cytofix Fixation Buffer (BD Biosciences), washed, incubated for 30 min at 4°C in Phosflow Perm Buffer III (BD Biosciences), washed again, and stained for 1 hr at room temperature with anti-pS6–Pacific Blue (clone D57.2.2E; Cell Signaling Technology).

### Peptides and tetramers

All peptides were synthesized at >95% purity (BioSynthesis Inc.). The EV20 peptide (YTAAEELAGIGILTVILGVL, Melan-A_21–40/A27L_) was used for *in vitro* priming studies. Fluorochrome-labeled tetrameric complexes of HLA-A*02:01–EV10 (ELAGIGILTV, Melan-A_26–35/A27L_) were generated in-house as described previously (15).

### In vitro priming of antigen-specific CD8^+^ T cells

Naive precursors specific for HLA-A2–EV10 were primed *in vitro* using an accelerated dendritic cell (DC) coculture protocol as described previously (11, 16, 17). Briefly, thawed PBMCs were resuspended at 5 × 10^6^ cells/well in AIM-V medium (Thermo Fisher Scientific) supplemented with Flt3 ligand (Flt3L; 50 ng/mL; R&D Systems) in the absence or presence of rosiglitazone (40 μM; Sigma-Aldrich) or IL-7 (20 ng/mL; R&D Systems). After 24 hr (day 1), the Melan-A peptide EV20 (1 μM) was added to the cultures, and DC maturation was induced using a standard cocktail of inflammatory cytokines, incorporating IL-1β (10 ng/mL), IL-7 (0.5 ng/mL), PGE2 (1 μM), and TNF (1,000 U/mL) (all from R&D Systems), or ssRNA40 (TLR8L; 0.5 μg/mL; InvivoGen). The cultures were supplemented on day 2 with FCS (10% v/v; Sigma-Aldrich). Medium was replaced every 3 days thereafter with fresh R+ containing FCS (10% v/v; Sigma-Aldrich). Antigen-specific CD8^+^ T cells were characterized via flow cytometry on day 10.

### RNA extraction and qPCR analysis

PBMCs were activated for 5 hr with plate-bound anti-CD3 (clone OKT3; Thermo Fisher Scientific). RNA was extracted from flow-sorted naive CD8^+^ T cells (n = 300 per condition) using a NucleoSpin RNA XS Kit (Macherey-Nagel), and cDNA was synthesized using Reverse Transcription Master Mix (Fluidigm). Specific targets were amplified using PreAmp Master Mix (Fluidigm). Gene expression was assessed using a BioMark HD System (Fluidigm) with EvaGreen Supermix (Bio-Rad). RNA expression levels were calculated using the 2^−ΔΔCT^ method with reference to a housekeeping gene (human 18S) (18).

### Statistics

Univariate statistical analyses were performed using nonparametric tests in Prism software version 8 (GraphPad Software Inc.). Unpaired groups were compared using the Mann-Whitney *U* test, and paired groups were compared using the Wilcoxon signed rank test. Correlations were determined using Spearman’s rank correlation. Significance was assigned at *p* < 0.05.

## Results

### Naive CD8^+^ T cells in the elderly exhibit altered basal activation status and proliferative capacity

**Figure 1:**
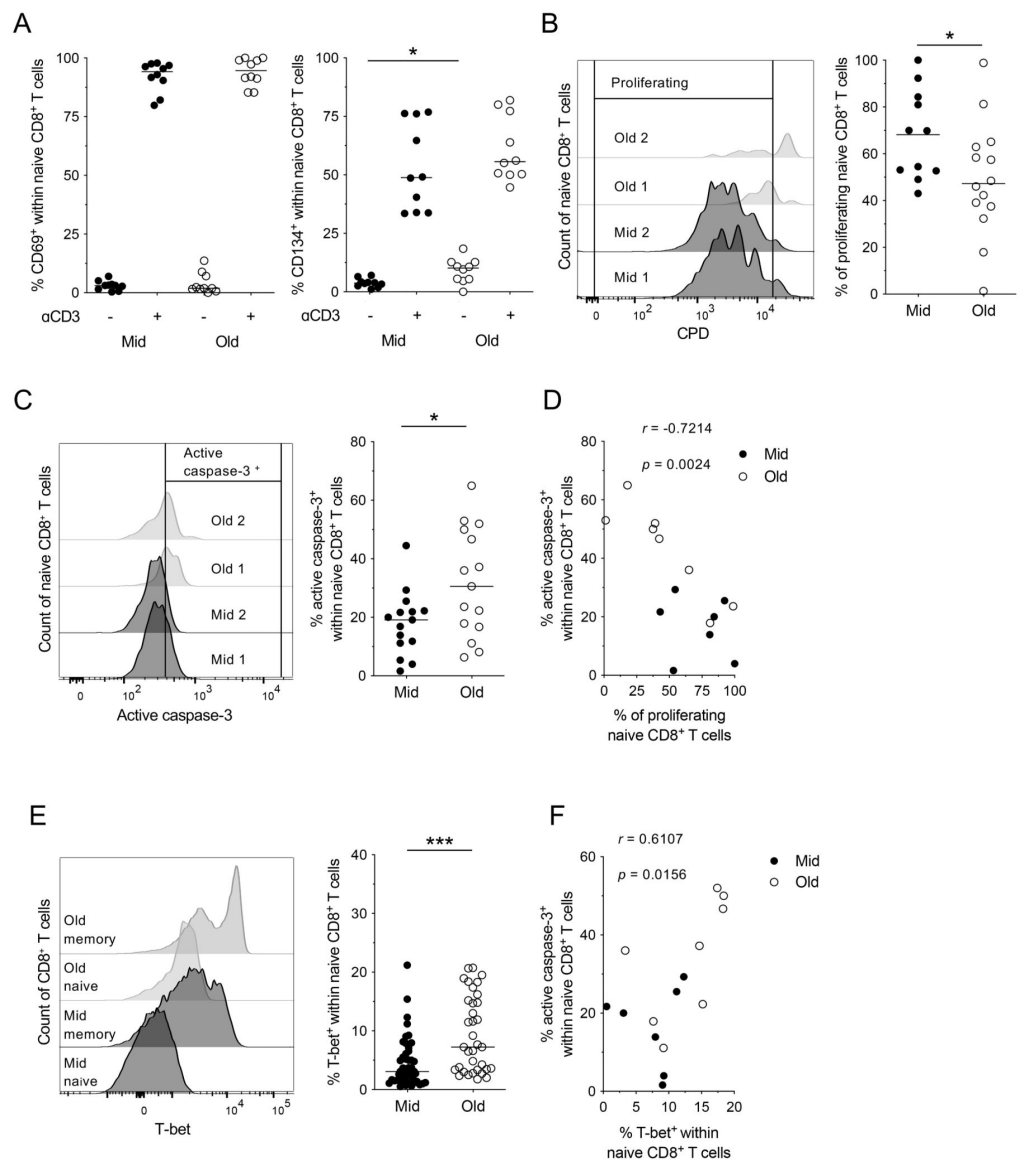
Activation and proliferation in the naive CD8^+^ T cell compartment. (**A**–**C**) PBMCs from middle-aged (Mid) and elderly individuals (Old) were incubated in the absence or presence of plate-bound anti-CD3. Surface expression of the activation markers CD69 and CD134 was measured after 24 hr (A), proliferation was measured after 4 days (B), and intracellular expression of active caspase-3 was measured after 1 day (C). Left panels: representative flow cytometry profiles. Right panels: data summaries. Data are shown for naive CD8^+^ T cells. Each dot represents one donor. Horizontal lines indicate median values. *p < 0.05 (Mann-Whitney *U* test), n = 10 (A), 11 (B) and 15 (C) for middle-aged and n = 10 (A), 14 (B) and 15 (C) for elderly donors. (**D**) Correlation between the frequency of naive CD8^+^ T cells that proliferated and the frequency of naive CD8^+^ T cells that expressed active caspase-3 after stimulation. Each dot represents one donor. Significance was determined using Spearman’s rank correlation, n = 7 for middle-aged and n = 8 for elderly donors. (**E**) T-bet expression was measured in unstimulated naive CD8^+^ T cells from middle-aged (Mid, n = 45) and elderly individuals (Old, n = 35). Left panel: representative flow cytometry profiles. Right panel: data summary. Each dot represents one donor. Horizontal lines indicate median values. ***p < 0.001 (Mann-Whitney *U* test). (**F**) Correlation between the basal expression frequency of T-bet and the activation-induced expression frequency of active caspase-3 among naive CD8^+^ T cells. Each dot represents one donor. Significance was determined using Spearman’s rank correlation, n = 7 for middle-aged and n = 8 for elderly donors.

In a previous study, we found that naive CD8^+^ T cells from elderly individuals responded suboptimally to cognate antigen stimulation, generating fewer effector memory CD8^+^ T cells (5). To extend these findings, we compared the activation profiles of naive CD8^+^ T cells from middle-aged and elderly individuals, mimicking antigen-driven signals with plate-bound anti-CD3. No age-related differences in activation *per se* were detected 24 hr after stimulation, as determined by measuring the upregulation of CD69 and CD134 (Figure 1A).

Despite these similarities, naive CD8^+^ T cells from elderly individuals proliferated to a lesser extent than naive CD8^+^ T cells from middle-aged individuals in response to stimulation (Figure 1B), confirming our earlier observations (5). We also found that activation-induced apoptosis was more common among naive CD8^+^ T cells from elderly *versus* middle-aged individuals, as determined by measuring the intracellular expression of active caspase-3 (Figure 1C). Of note, there was a strong inverse correlation between the frequency of naive CD8^+^ T cells that proliferated and the frequency of naive CD8^+^ T cells that expressed active caspase-3 after stimulation, irrespective of age (Figure 1D).

It was also notable that unstimulated naive CD8^+^ T cells from elderly individuals expressed CD134 more commonly than unstimulated naive CD8^+^ T cells from middle-aged individuals, consistent with elevated levels of basal activation (Figure 1A). To corroborate this observation, we measured the expression of T-bet, which is classically upregulated in response to activation via the TCR. The basal expression frequencies of T-bet mirrored the basal expression frequencies of CD134 (Figure 1E). Equivalent results were obtained using a more stringent definition of naive CD8^+^ T cells (Figures S1A and S1B), which excluded phenotypically similar memory CD8^+^ T cells (19). Moreover, the basal expression frequency of T-bet correlated directly with the activation-induced expression frequency of active caspase-3 among naive CD8^+^ T cells, irrespective of age (Figure 1F).

Collectively, these data revealed that elevated levels of basal activation and a predisposition to apoptosis were associated with an age-related deficit in the proliferative capabilities of naive CD8^+^ T cells, despite a largely unaltered response to activation via the TCR.

### Naive CD8^+^ T cells in the elderly are metabolically distinct

**Figure 2:**
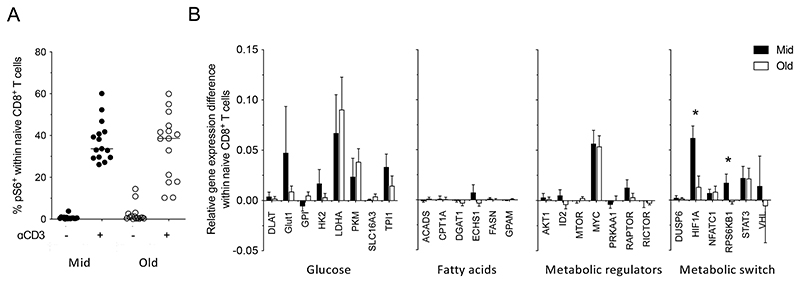
Activation-induced metabolic changes in the naive CD8^+^ T cell compartment. (**A**) PBMCs from middle-aged (Mid, n = 15) and elderly individuals (Old, n = 15) were incubated in the absence or presence of plate-bound anti-CD3. Intracellular expression of the mTOR activity marker pS6 was measured after 3 hr. Data are shown for naive CD8^+^ T cells. Each dot represents one donor. Horizontal lines indicate median values. *p < 0.05 (Mann-Whitney *U* test) (**B**) Flow-sorted naive CD8^+^ T cells from middle-aged (black bars; n = 4) and elderly individuals (white bars; n = 5) were incubated in the absence or presence of plate-bound anti-CD3. Gene expression levels were measured after 5 hr. Data are shown relative to the unstimulated condition. Bars indicate mean ± SEM. *p < 0.05 (Mann-Whitney *U* test).

Signals transduced via the TCR elicit an mTOR-driven metabolic switch that supports the function and viability of activated naive CD8^+^ T cells (11). We therefore assessed mTOR activity by quantifying phospho-S6 (pS6). In line with the comparable activation profiles, naive CD8^+^ T cells from middle-aged and elderly individuals upregulated mTOR activity to a similar extent after stimulation with plate-bound anti-CD3 (Figure 2A). To validate these findings, we measured the expression of metabolism-related genes, comparing unstimulated and activated naive CD8^+^ T cells. Genes encoding various enzymes involved in glycolysis were upregulated similarly in flow-sorted naive CD8^+^ T cells from middle-aged and elderly individuals after stimulation with plate-bound anti-CD3 (Figure 2B). In contrast, genes associated with lipid metabolism or signaling pathways involved in metabolic regulation were not generally upregulated in response to stimulation, with the exception of *MYC*, which was overexpressed in activated naive CD8^+^ T cells, irrespective of age (Figure 2B). Genes that play a critical role in the metabolic switch were also overexpressed in activated naive CD8^+^ T cells, irrespective of age, with the exception of *HIF1* and *RPS6KB1,* which were upregulated to a greater extent in activated naive CD8^+^ T cells from middle-aged *versus* elderly individuals (Figure 2B). These data support a rather intact although suboptimal metabolic switch, with a physiological mTOR activation, in naive CD8^+^ T cells of elderly subjects upon TCR ligation.

**Figure 3:**
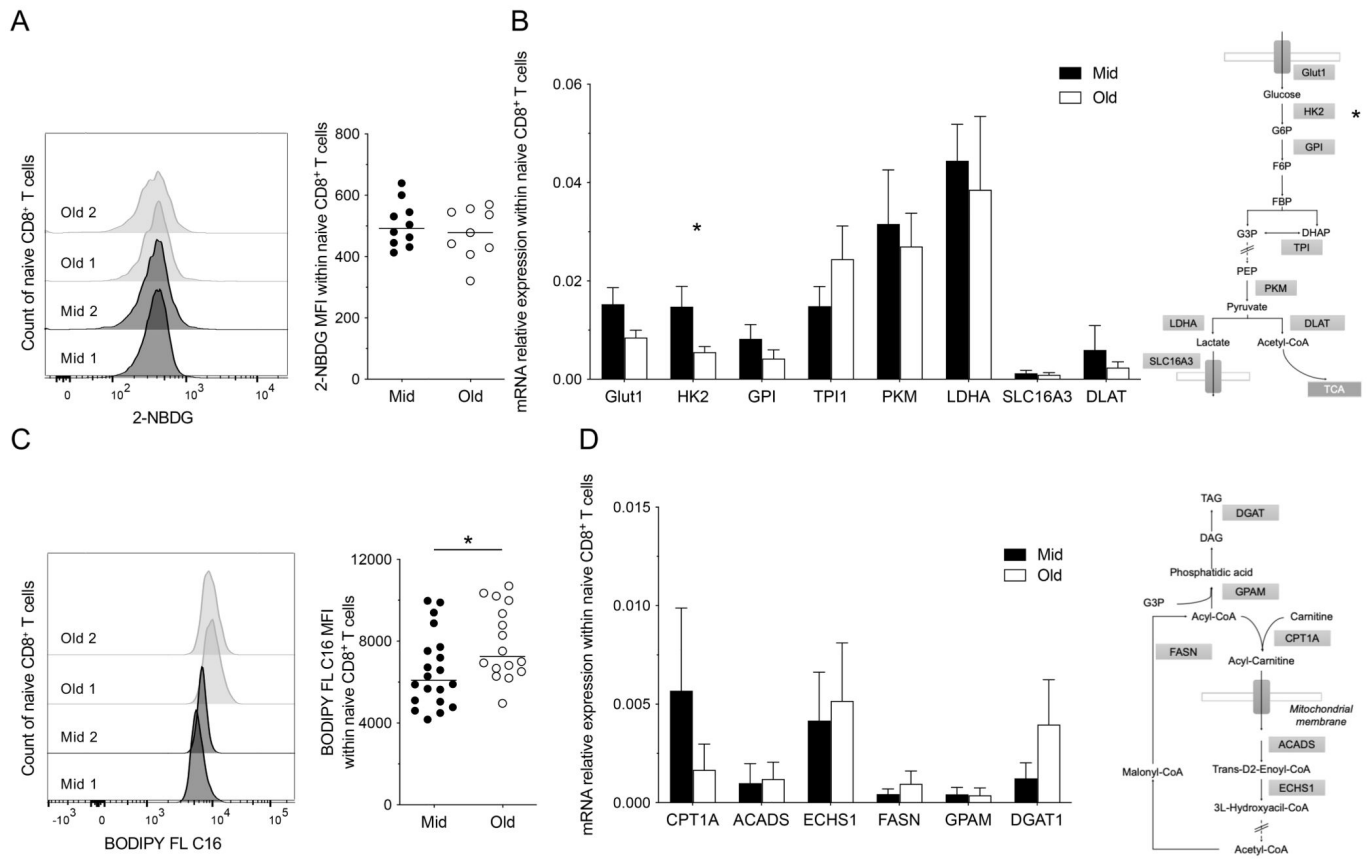
Basal metabolism in the naive CD8^+^ T cell compartment. (**A** & **C**) Glucose (A) and FA uptake (C) were measured in unstimulated naive CD8^+^ T cells from middle-aged (Mid) and elderly individuals (Old) by determining the mean fluorescence intensity (MFI) of 2-NBDG and BODIPY FL C16, respectively. Left panels: representative flow cytometry profiles. Right panels: data summaries. Each dot represents one donor. Horizontal lines indicate median values. *p < 0.05 (Mann-Whitney *U* test), n = 10 (A) and 20 (C) for middle-aged and n = 9 (A) and 16 (C) for elderly donors. (**B** & **D**) Expression levels of genes related to glucose (B) and FA metabolism (D) were measured in unstimulated naive CD8^+^ T cells flow-sorted from middle-aged (black bars; n = 5) and elderly individuals (white bars; n = 5). Data are shown relative to 18S. Bars indicate mean ± SEM. *p < 0.05 (Mann-Whitney *U* test).

To explore these age-related phenomena in more depth, we investigated the metabolic and transcriptional properties of quiescent naive CD8^+^ T cells. Glycolysis is the main metabolic pathway that supports the activation of naive CD8^+^ T cells (11, 20). We found no significant differences in basal glucose uptake between unstimulated naive CD8^+^ T cells from middle-aged individuals and unstimulated naive CD8^+^ T cells from elderly individuals (Figure 3A). Moreover, we found similar basal expression levels of glycolysis-related genes, with the exception of *HK2*, which was overexpressed in unstimulated naive CD8^+^ T cells from middle-aged *versus* elderly individuals (Figure 3B). This gene encodes a selectively regulated isoform of hexokinase (21), which catalyzes glucose phosphorylation and is usually induced in response to stimulation via the TCR (21, 22).

**Figure 4:**
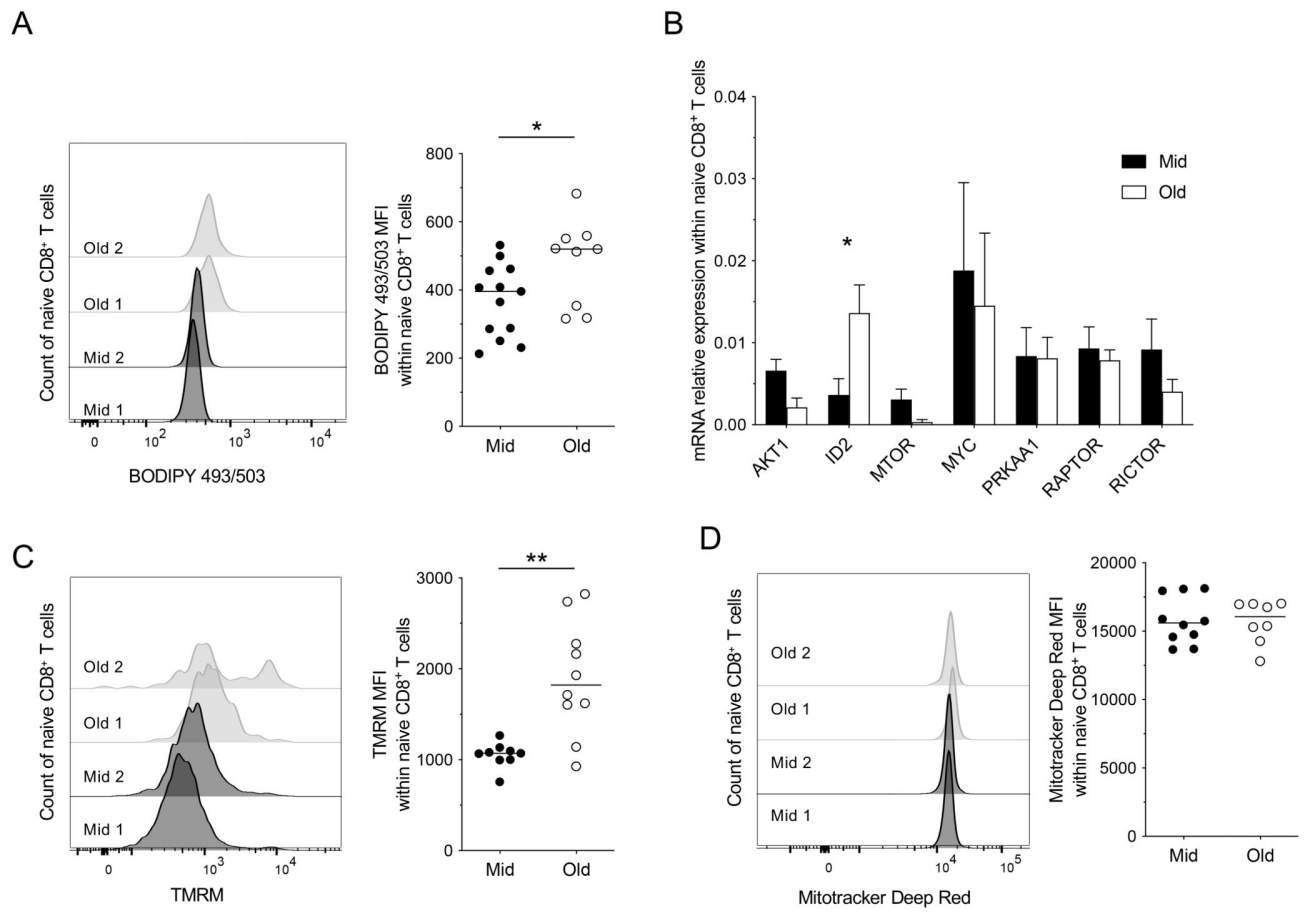
Metabolic regulation in the naive CD8^+^ T cell compartment. (**A**, **C**, & **D**) NL content (A), ΔΨM (C), and mitochondrial mass (D) were measured in unstimulated naive CD8^+^ T cells from middle-aged (Mid) and elderly individuals (Old) by determining the mean fluorescence intensity (MFI) of BODIPY 493/503, TMRM, and Mitotracker Deep Red, respectively. Left panels: representative flow cytometry profiles. Right panels: data summaries. Each dot represents one donor. Horizontal lines indicate median values. *p < 0.05, **p < 0.01 (Mann-Whitney *U* test), n = 13 (A), 9 (C) and 10 (D) for middle-aged and n = 9 (A), 10 (C) and 8 (D) for elderly donors. (**B**) Expression levels of genes related to signaling pathways involved in metabolic regulation were measured in unstimulated naive CD8^+^ T cells flow-sorted from middle-aged (black bars; n = 5) and elderly individuals (white bars; n = 5). Data are shown relative to 18S. Bars indicate mean ± SEM. *p < 0.05 (Mann-Whitney *U* test).

In contrast, FA uptake was increased among unstimulated naive CD8^+^ T cells from elderly *versus* middle-aged individuals (Figure 3C), although this difference was not associated with significant changes in the expression levels of genes encoding various enzymes involved in FA synthesis or FA oxidation (FAO). However, we noted that *DGAT1,* which encodes diacylglycerol O-acyltransferase 1, a key enzyme involved in the storage of FAs as triacylglycerol (TAG), was expressed at higher levels in unstimulated naive CD8^+^ T cells from elderly *versus* middle-aged individuals, albeit without reaching statistical significance (Figure 3D). Unstimulated naive CD8^+^ T cells from elderly individuals also stored higher amounts of NLs than unstimulated naive CD8^+^ T cells from middle-aged individuals (Figure 4A).

In further experiments, we assessed the basal expression levels of various genes encoding transcription factors involved in metabolic regulation. Consistent patterns of expression were observed in unstimulated naive CD8^+^ T cells, irrespective of age, with the exception of *ID2*, which was expressed at higher levels in unstimulated naive CD8^+^ T cells from elderly *versus* middle-aged individuals (Figure 4B). *ID2* is involved in metabolic adaptation (23, 24) and promotes lipid storage via the downmodulation of *PGC-1α* (24), which enhances FAO and inhibits TAG synthesis (25). Moreover, *ID2* promotes an overall increase in ΔΨM, without affecting mitochondrial biogenesis or, by extension, mitochondrial mass (23). In line with these known functions, ΔΨM was higher in unstimulated naive CD8^+^ T cells from elderly *versus* middle-aged individuals (Figure 4C), whereas mitochondrial mass was largely unaffected by age (Figure 4D). We also noted a direct correlation between ΔΨM and the frequency of unstimulated naive CD8^+^ T cells that expressed T-bet, suggesting a link with the loss of quiescence (Figure S2).

Collectively, these data revealed an age-related shift in the basal metabolic properties of naive CD8^+^ T cells, typified by high levels of FA uptake and NL storage and a supranormal ΔΨM.

### Naive CD8^+^ T cells in the elderly can be reinvigorated with lipid-altering drugs

T cell homeostasis and viability can be affected by high levels of FAs (26, 27). In line with this paradigm, we found that bulk CD8^+^ T cells from middle-aged donors exhibited higher ΔΨM values and more commonly expressed T-bet after treatment with increasing amounts of PA (Figures S3A–B). High PA doses (3000 μM) also induced T-cell death (Figure S3C). Moreover, these changes were associated with impaired proliferative responses (Figure S3D), mimicking the altered physiology of naive CD8^+^ T cells from elderly individuals and suggesting a determinative role for FAs in these age-related transitions.

**Figure 5:**
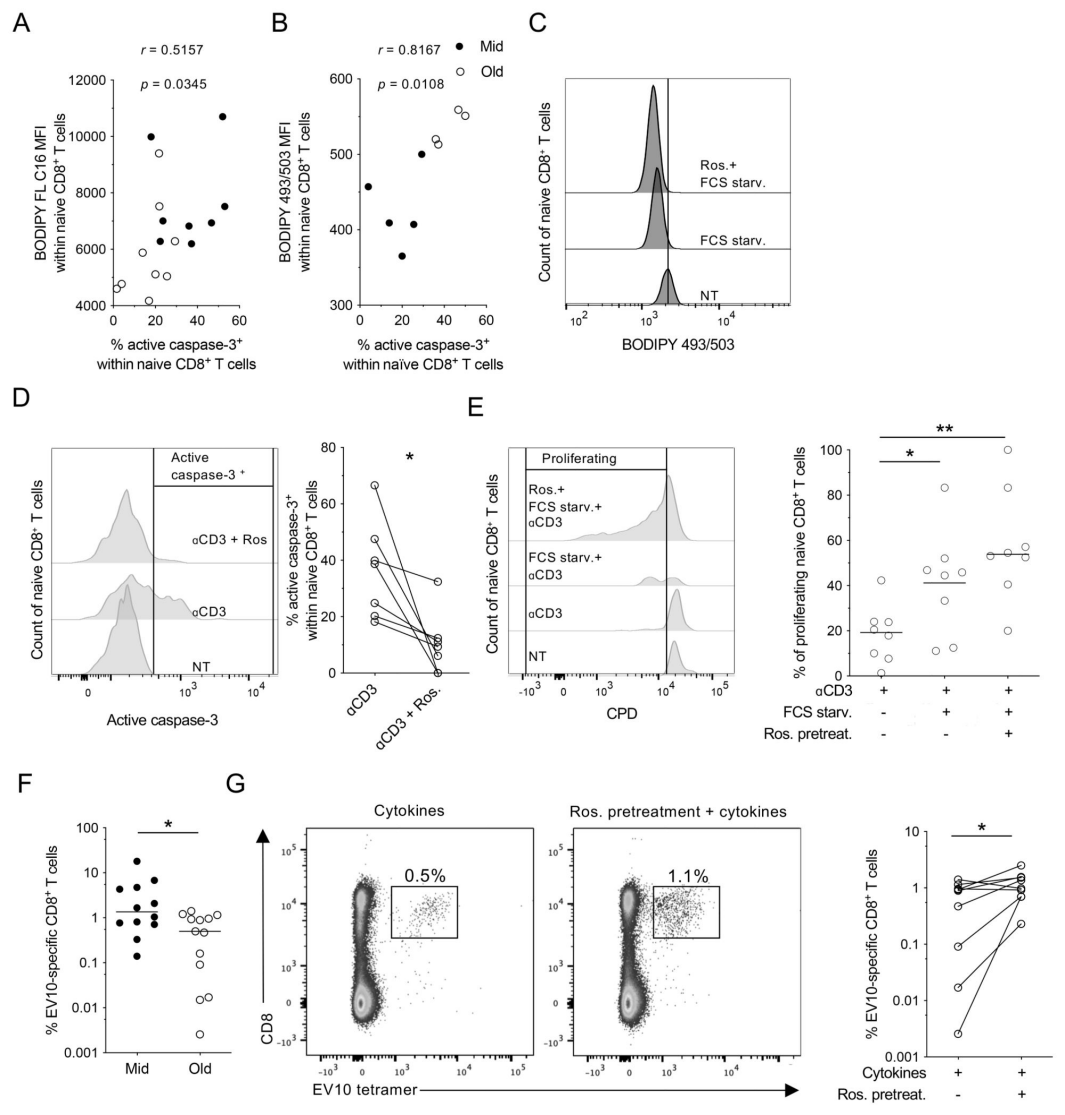
Effects of lipid-altering drugs in the naive CD8^+^ T cell compartment. (**A** & **B**) Correlations between the frequency of unstimulated naive CD8^+^ T cells that expressed active caspase-3 and basal levels of FA uptake (A) and NL content (B) measured by determining the mean fluorescence intensity (MFI) of BODIPY FL C16 and BODIPY 493/503, respectively. Each dot represents one donor. Significance was determined using Spearman’s rank correlation, n = 9 (A) and 5 (B) for middle-aged and n = 8 (A) and 4 (B) for elderly donors. (**C**) PBMCs were preincubated for 2 days in serum-free medium in the absence or presence of rosiglitazone (Ros). NL content was measured in naive CD8^+^ T cells by determining the mean fluorescence intensity (MFI) of BODIPY 493/503. Flow cytometry profiles are representative of five independent experiments. (**D**) PBMCs from elderly individuals (n = 8) were stimulated with plate-bound anti-CD3 in the absence or presence of rosiglitazone (Ros). Active caspase-3 expression was measured after 24 hr. Data are shown for naive CD8^+^ T cells. Left panel: representative flow cytometry profiles. Right panel: data summary. Each dot represents one donor. *p < 0.05 (Wilcoxon signed rank test). (**E**) PBMCs from elderly individuals (n = 8) were preincubated for 2 days in serum-free medium in the absence or presence of rosiglitazone (Ros) and stimulated with plate-bound anti-CD3. Proliferation was measured after 4 days. Data are shown for naive CD8^+^ T cells. Left panel: representative flow cytometry profiles. Right panel: data summary. Each dot represents one donor. Horizontal lines indicate median values. *p < 0.05, **p < 0.01 (Mann-Whitney *U* test). (**F**) Percentage of tetramer^+^ EV10-specific CD8^+^ T cells expanded from middle-aged (Mid) and elderly individuals (Old) for 10 days in the presence of Flt3L and a cocktail of inflammatory cytokines. Each dot represents one donor. Horizontal lines indicate median values. *p < 0.05 (Mann-Whitney *U* test), n = 12 for middle-aged and n = 13 for elderly donors. (**G**) Percentage of tetramer^+^ EV10-specific CD8^+^ T cells expanded from elderly individuals (n = 9) for 10 days in the presence of Flt3L and a cocktail of inflammatory cytokines after preincubation for 2 days in the absence or presence of rosiglitazone (Ros). Left panel: representative flow cytometry profiles. Right panel: data summary. Each dot represents one donor. Horizontal lines indicate median values. *p < 0.05 (Wilcoxon signed rank test). NT: not treated.

In further experiments, we observed a direct correlation between the frequency of unstimulated naive CD8^+^ T cells that expressed active caspase-3 and the corresponding basal levels of FA uptake (Figure 5A) and NL content (Figure 5B). To determine the biological relevance of these associations, we treated naive CD8^+^ T cells with rosiglitazone, a drug known to foster lipid catabolism by activating triglyceride lipase (28) and preventing the conversion of FAs into NLs (29). As expected, NL content was reduced after exposure to rosiglitazone, consistent with enhanced catabolism, and similar effects were observed after serum starvation, consistent with forced intracellular consumption (Figure 5C). Pretreatment with rosiglitazone also inhibited activation-induced apoptosis among naive CD8^+^ T cells from elderly individuals (Figure 5D), and similar results were obtained using fenofibrate, which induces lipid catabolism by enhancing FAO (Figure S3E).

Importantly, naive CD8^+^ T cells from elderly individuals proliferated to a greater extent after serum starvation, and the addition of rosiglitazone further enhanced these activation-induced proliferative responses (Figure 5E). To assess the potential relevance of these findings in the context of antigen-driven immune responses, we used an *in vitro* model to prime naive CD8^+^ T cells specific for the melanoma-associated epitope EV10 (5). We found that EV10-specific CD8^+^T cells from middle-aged individuals expanded to a greater extent than EV10-specific CD8^+^ T cells from elderly individuals (Figure 5F), which could be due to both a higher frequency of precursors at younger age or to an age-related deficit in proliferation. In line with this latter interpretation, preincubation with rosiglitazone enhanced the expansion of EV10-specific CD8^+^ T cells from elderly individuals (Figure 5G), mirroring the results obtained with plate-bound anti-CD3.

Collectively, these data revealed that age-related functional deficits associated with abnormal lipid metabolism and greater levels of basal activation in the naive CD8^+^ T cell compartment may be, at least in part, reversed in the presence of rosiglitazone, highlighting a new therapeutic approach that could enhance immune reactivity against newly encountered antigens in the elderly population.

## Discussion

A detailed understanding of age-related deficits in the naive T cell compartment is essential for the rational development of immunotherapies and vaccines that protect elderly individuals from emerging threats, such as COVID-19. We found that naive CD8^+^ T cells from elderly individuals were susceptible to apoptosis and proliferated suboptimally in response to stimulation via the TCR. These abnormalities were associated with enhanced levels of basal activation, measured in terms of ΔΨM and the *ex vivo* expression frequencies of T-bet and CD134. Although characteristic of advanced age, it may be of interest to investigate if some functional and metabolic alterations may already occur in pre-elderly subjects (i.e. in individuals <65y).

Recent studies have shown that metabolic processes govern the behavior of T cells (11, 20, 30, 31). In the naive CD8^+^ T cell compartment, autophagy and glycolysis are typically upregulated in response to activation (11, 32, 33), whereas homeostatic energy requirements are fulfilled primarily via FAO (13, 34–37). This metabolic switch was largely unaffected by age in our study, but at rest, naive CD8^+^ T cells from elderly individuals displayed abnormally high levels of FA uptake and stored abnormally high amounts of NLs.

In line with previous reports suggesting that excessively high levels of intracellular lipids may be toxic (11, 26, 38), we found that active caspase-3 expression correlated directly with FA uptake and NL content in the naive CD8^+^ T cell compartment. Lipids are essential for T cell activation and proliferation (39). Supraphysiological amounts of intracellular lipids can nonetheless impair T cell proliferation and viability (40–42). Accordingly, we found that activation-induced initiation of the apoptotic pathway was reduced by interventions that enhanced lipid clearance in naive CD8^+^ T cells. Of note, NLs *per se* are not toxic. The conversion of FAs into NLs therefore most likely protects against lipotoxicity under homeostatic conditions (26), although further studies are warranted to investigate the molecular linkage between altered T-cell metabolism and increased susceptibility to apoptosis with ageing.

The heightened basal activation status of naive CD8^+^ T cells from elderly individuals seemed to be sustained energetically by increased mitochondrial activity, given that T-bet expression correlated directly with ΔΨM. Inflammation is closely linked with metabolic dysregulation in the elderly (43). High levels of circulating proinflammatory cytokines and lipids are common features of advanced age and may contribute to the disruption of cellular quiescence. Moreover, hematopoietic progenitor cells in elderly individuals are often metabolically active, and this trait may be heritable (44). Increased rates of homeostatic proliferation are required to maintain naive CD8^+^ T cell numbers in the elderly (45), and the predominant energetic pathway that supports this process is thought to be FAO (46, 47). It is therefore plausible that high basal levels of FA uptake and NL storage constitute a bioenergetic pattern that favors homeostatic proliferation.

Aging is characterized by profound metabolic perturbations (48), including increased lipogenesis (49) and reduced lipolysis (50), leading to higher systemic levels of free FAs and TAG (43, 51). The combination of a homeostatic environment and high systemic levels of proinflammatory cytokines and lipids may therefore underlie the altered metabolism and functional deficits that characterize naive CD8^+^ T cells in the elderly. A key finding of our study was the observation that rosiglitazone, a drug known to foster lipid catabolism, largely reversed these abnormalities and enhanced antigen-driven CD8^+^ T cell responses in an experimental model that has been shown to recapitulate *de novo* priming events *in vivo* (5, 52, 53). Of note, rosiglitazone was recently found to attenuate the metabolic phenomena associated with advanced age and extend longevity in mice (54), and thiazolidinediones in general may have other beneficial effects on the immune system (55). Moreover, drugs that enhance FAO, such as fibrates, appear to enhance the quality and quantity of effector CD8^+^ T cells (11, 56–58). These are encouraging results from a translational perspective, although it should be noted that rosiglitazone has now been withdrawn as a therapeutic agent in Europe. Our data have nonetheless provided an important proof-of-principle demonstration suggesting that lipid-altering drugs could be helpful as adjunctive interventions to enhance adaptive immune responses against previously unencountered antigens, particularly in elderly individuals, who often respond poorly to vaccination and remain vulnerable to emerging pathogens, such as seasonal influenza viruses and SARS-CoV-2.

## Supplementary Material

Supplementary Material

## Key points

- Naive CD8^+^ T cells from elderly humans are characterized by functional alterations
- Old naive T-cell functional impairment is linked to altered basal lipid metabolism
- Lipid-lowering approaches can improve old naive T-cell functions *in vitro*
