# Supplementary material for: Altered basal lipid metabolism underlies the functional impairment of naive CD8+ T cells in elderly humans
Source: J Immunol. Author manuscript; Available in PMC 2023 Oct 6. (PMC7615155; doi:10.4049/jimmunol.2100194)
Supplement: Supplementary Material [file EMS181810-supplement-Supplementary_Material.docx]

**SUPPLEMENTARY MATERIAL**

**Figure S1. T-bet expression in naive CD8^+^ T cells.** (**A**) Flow cytometric gating strategy used to exclude memory CD8^+^ T cells with a naive phenotype (MNP). (**B**) T-bet expression in naive CD8^+^ T cells (CD3^+^ CD8^+^ CD27^+^ CD45RA^+^ CCR7^+^ CD49d^−^ CD57^−^ CD95^−^) from middle-aged (Mid) and elderly individuals (Old). Each dot represents one donor. Horizontal lines indicate median values. **p* < 0.05 (Mann-Whitney *U* test).

**Figure S2. Correlation between T-bet expression and mitochondrial membrane potential** **in naive CD8^+^ T cells.** Correlation between the frequency of unstimulated naive CD8^+^ T cells that expressed T-bet and basal ΔΨM, measured by determining the mean fluorescence intensity (MFI) of TMRM. Each dot represents one donor. Significance was determined using Spearman's rank correlation.

**Figure S3. *In vitro* modulation of CD8^+^ T cell metabolism.** (**A**, **B**) PBMCs from middle-aged individuals were cultured for 5 hr in the absence or presence of the indicated doses of palmitic acid (PA). T-bet expression (A) and ΔΨM (B) were measured in bulk CD8^+^ T cells via intracellular staining or by determining the mean fluorescence intensity (MFI) of TMRM, respectively. Each dot represents one donor. Horizontal lines indicate median values. **p* < 0.05, ***p* < 0.01 (Wilcoxon signed rank test with Bonferroni correction). (**C**) PBMCs from middle-aged individuals (n = 7) were cultured in the absence or presence of the indicated doses of palmitic acid (PA) for up to 48 hr without stimulation or for 4 days after stimulation with plate-bound anti-CD3. The proportion of live cells was measured via flow cytometry. Bars indicate mean ± SEM. (**D**) PBMCs from middle-aged individuals were preincubated for 1 day in serum-free medium in the absence or presence of palmitic acid (PA) and then stimulated with plate-bound anti-CD3. Proliferation was measured after 4 days. Data are shown for bulk CD8^+^ T cells. Each dot represents one donor. Horizontal lines indicate median values. **p* < 0.05 (Wilcoxon signed rank test). (**E**) PBMCs from elderly individuals were stimulated with plate-bound anti-CD3 in the absence or presence of fenofibrate (Fen). Active caspase-3 expression was measured after 24 hr. Data are shown for naive CD8^+^ T cells. Left panel: representative flow cytometry profiles. Right panel: data summary. Each dot represents one donor. **p* < 0.05 (Wilcoxon signed rank test). NT: not treated.
